# The molecular mechanisms of human separase regulation

**DOI:** 10.1042/BST20221400

**Published:** 2023-05-04

**Authors:** Jun Yu, David O. Morgan, Andreas Boland

**Affiliations:** 1 Department of Molecular and Cellular Biology, University of Geneva, CH-1211 Geneva, Switzerland; 2 Department of Physiology, University of California, San Francisco, San Francisco, CA 94143, U.S.A.

**Keywords:** cancer, cell cycle, cryo-electron microscopy, enzyme–substrate interactions, inhibition, structural biology

## Abstract

Sister chromatid segregation is the final irreversible step of mitosis. It is initiated by a complex regulatory system that ultimately triggers the timely activation of a conserved cysteine protease named separase. Separase cleaves the cohesin protein ring that links the sister chromatids and thus facilitates their separation and segregation to the opposite poles of the dividing cell. Due to the irreversible nature of this process, separase activity is tightly controlled in all eukaryotic cells. In this mini-review, we summarize the latest structural and functional findings on the regulation of separase, with an emphasis on the regulation of the human enzyme by two inhibitors, the universal inhibitor securin and the vertebrate-specific inhibitor CDK1–cyclin B. We discuss the two fundamentally different inhibitory mechanisms by which these inhibitors block separase activity by occluding substrate binding. We also describe conserved mechanisms that facilitate substrate recognition and point out open research questions that will guide studies of this fascinating enzyme for years to come.

## Introduction

During mitosis, replicated chromosomes are aligned on the mitotic spindle and segregated equally to the daughter cells. To ensure correct segregation, sister chromatid pairs are linked during S phase [1,2] by the ring-shaped protein complex cohesin [3,4], which topologically entraps the sister chromatids [5]. In late metaphase, the cohesin subunit Scc1/Rad21 [6–8] is cleaved by separase, an evolutionarily conserved cysteine protease, to allow separation and segregation of sister chromatids in anaphase [6,7,9]. Separase also cleaves the meiotic cohesin subunit Rec8 [10], as well as unrelated proteins such as Meikin [11], MCL1 and BCL-XL [12], and Pericentrin/Kendrin [13–15], and is, therefore, believed to have functions beyond cohesin cleavage, as described elsewhere [16,17].

Premature activation of separase leads to genomic instability and aneuploidy, which can lead to tumorigenesis [18–20]. Consequently, the proteolytic activity of human separase is regulated during mitosis through mutually exclusive binding to two inhibitory binding partners, namely securin [21–24] or the CDK1–cyclin B complex [25–27]. Furthermore, the human SGO2 (shugoshin 2)–MAD2 complex is thought to act as a securin-independent inhibitor prior to mitosis and during a spindle assembly checkpoint arrest [28]. While securin is a universal inhibitor of separase that is conserved in yeast and humans and binds separase co-translationally [29], regulation of separase through binding to CDK1–cyclin B is vertebrate-specific [25,27,29,30].

**Figure 1.**
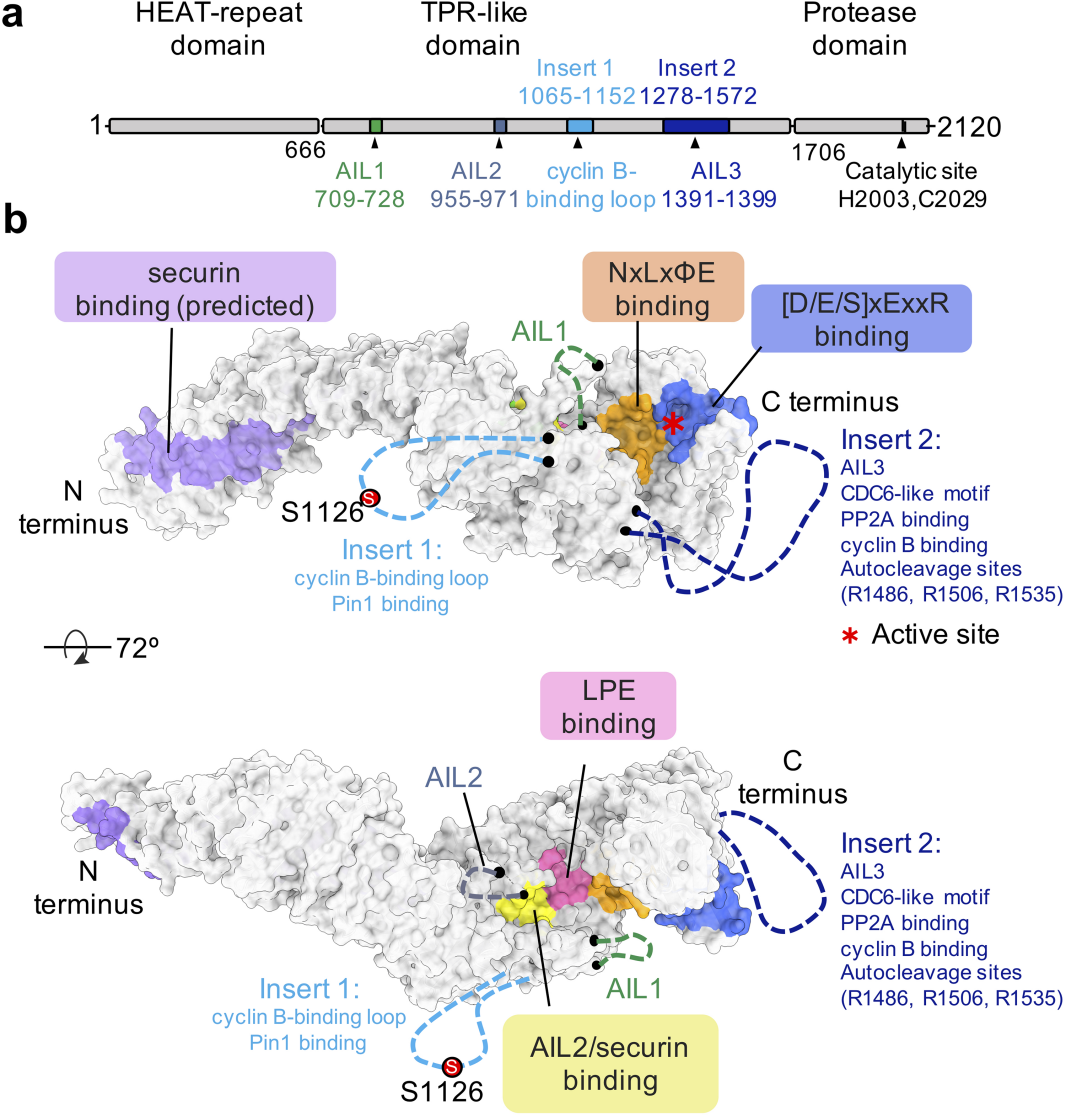
Domains of human separase and binding sites of separase-interacting proteins. (**a**) Human separase is 2120 aa and consists of three domains: A HEAT-repeat domain, a TPR-like domain, and a protease domain. Two large flexible loops, insert 1 (aa 1065–1152) and insert 2 (aa 1278–1572), are shown with blocks in cyan and deep blue, respectively. The three auto-inhibitory loops (AIL1–3) are located in the TPR-like domain and indicated by triangles; AIL3 is part of insert 2. The catalytic site is composed of the highly conserved His2003 and Cys2029 residues. (**b**) Human separase has multiple binding partners and binding sites are indicated as surface representation (PDB code: 7NJ1). In the top diagram, the putative securin (aa 163–202) binding site in the N-terminal HEAT-repeat domain is predicted by AlphaFold2 [43] and shown in purple. Binding sites for the NxLxΦE and (S/D/E)xExxR motifs, on the C-terminal protease domain, are close to the active site marked with a star symbol. Insert 1 contains the cyclin B-binding site, including phosphorylated serine 1126 and a possible Pin1 binding site. Insert 2 contains AIL3, a CDC6-like motif that binds CDK1, and a PP2A binding site near the autocleavage sites. In the bottom diagram, AIL2 and LPE motif binding sites are indicated in yellow and pink, respectively. AIL1 and AIL2, insert 1 and insert 2 (including AIL3) are shown with dashed lines in dark green, steel blue, cyan and deep blue.

Human separase consists of 2120 amino acids (aa), with a molecular mass of 233 kDa. The enzyme can be divided structurally into three domains: an N-terminal HEAT-repeat (huntingtin, elongation factor 3 (EF3), protein phosphatase 2A (PP2A), and TOR1) domain, followed by a tetratricopeptide repeat (TPR)-like domain and a conserved C-terminal protease domain that is responsible for substrate cleavage [30–33]. Two large, intrinsically disordered insertions (insert 1 and insert 2) emerge from the central TPR-like domain. Both inserts mediate protein–protein interactions and are important for the regulation of human separase activity. A schematic depiction of the domain architecture and two surface representations that indicate the binding sites of separase-interacting proteins are shown in Figure 1. Insert 1 (also termed the cyclin B-binding loop) interacts with cyclin B1 [25,27,30] and possibly the peptidyl-prolyl-isomerase Pin1 [34]. Insert 2 contains a CDC6-like motif that binds cyclin-dependent kinase 1 (CDK1) [27,30], as well as other motifs that bind PP2A [35], cyclin B1 and separase itself [30]. Insert 2 also contains three separase autocleavage sites that are adjacent to the PP2A-binding region [24,35] (Figure 1b). Upon self-cleavage, the N- and C-terminal fragments of separase remain stably attached and the proteolytic function of the protein is not affected [24,36]. However, separase self-cleavage disrupts PP2A binding and possibly promotes the binding of separase to the CDK1–cyclin B1 complex *in vivo* [35,37].

In this review, we focus on the latest structural and functional findings on separase substrate recognition, the diverse modes of proteolytic inhibition by securin and the CDK1–cyclin B1–Cks1 (CCC) complex, regulation of the protease activity by vertebrate-specific binding partners, and the implications of the latest discoveries for ordered cell cycle progression.

## Substrate recognition by separase

Structural studies of the separase protease domain fused to a substrate-mimicking peptide [31], as well as multiple studies of full-length separase bound to the inhibitory pseudosubstrate securin [30,32,33], have provided important insights into substrate recognition and the substrate-induced cleavage mechanism of separase. In addition, numerous biochemical studies have helped to define key principles important for high-affinity substrate binding and efficient cleavage.

A common feature of all identified separase substrates is the unstructured nature of the polypeptide N- and C-terminal of the cleavage site, which allows the insertion of the cleavage motif into the catalytic site of separase [31,38]. Separase substrates are cleaved immediately C-terminal of an arginine residue at the P1 cleavage position within an (S/D/E)xExxR cleavage motif (called the P6–P1 positions) [7,8,38]. Interactions between the P1 Arg and separase residues lining the catalytic pocket orchestrate the active configuration of the catalytic site, explaining the strict dependency on a P1 Arg residue to promote substrate-induced cleavage [31,33]. More precisely, a conserved aspartate residue in the catalytic pocket forms a bidentate salt bridge with the guanidinium group of the substrate P1 arginine. This interaction facilitates hydrogen bonding between the Nε atom of the guanidinium group of the arginine and the main chain carbonyl of a conserved glycine residue in separase, adjacent to the catalytic histidine side chain. Precise positioning of the catalytic histidine allows the creation of the oxyanion hole necessary for the cleavage of the scissile peptide bond [31].

**Figure 2.**
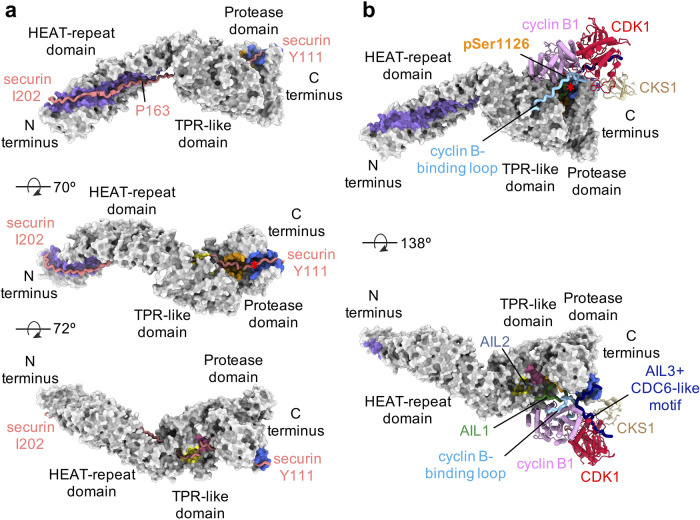
Structures of human separase bound to securin or CDK1–cyclin B1–CKS1 (CCC). (**a**) Three different views showing securin (aa 111–202) binding to separase in an antiparallel fashion (PDB code: 7NJ1). Separase is shown as surface representation in gray and securin is shown as cartoon in salmon. Securin (aa 163–202) binding to separase (aa 1–443) is predicted by AlphaFold2. The active site of separase is highlighted by a star in the middle diagram. Binding sites of separase-interacting proteins are color coded as in Figure 1b. (**b**) Two views show CCC binding to the TPR-like domain and C-terminal protease domain of separase (PDB code: 7NJ0). Cyclin B-binding loop (insert 1; cyan), containing phosphorylated serine 1126 (orange), wraps around cyclin B1. AIL3 binds to separase at the (S/D/E)xExxR motif binding site adjacent to the active site, while the CDC6-like motif binds the CDK1 active site as a pseudosubstrate. AIL1, containing the NxLxΦE motif, becomes ordered and binds to separase in a cleft between the TPR-like domain and the protease domain. AIL2 binds to separase at a hydrophobic site.

**Figure 3.**
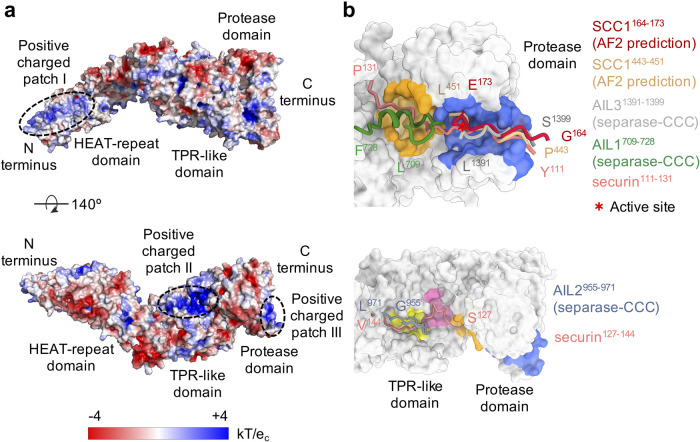
Electrostatic surface potential of human separase and comparison of securin, Scc1 and AIL1-3 binding to separase. (**a**) The electrostatic surface potential of separase was calculated using APBS [58] in PyMOL [59] (PDB code: 7NJ1). Three large positively charged patches (I–III) are indicated with dashed ovals. (**b**) Securin binds to separase at multiple sites, including the (S/D/E)xExxR motif binding site (blue), the NxLxΦE motif binding site (orange), the LPE motif binding site (pink) and the hydrophobic site (yellow). AIL3 and Scc1 fragments (residues 164–173 or 443–451) that include cleavage sites bind to separase at the (S/D/E)xExxR motif binding site (blue), similarly to securin. AIL1 contains a NDLNYE motif forming a small α-helix and binds to separase at the NxLxΦE motif binding site (orange). In the absence of securin, AIL2 becomes rigid and occupies the hydrophobic site (yellow). The binding of Scc1 to separase was predicted by AlphaFold2. Figures have been generated using Chimera X [60] or the PyMOL Molecular Graphics System.

In substrates, additional separase-binding motifs have recently been identified outside the conserved cleavage motif in Scc1 and are crucial facilitators of high-affinity binding and regulation of proteolysis [30,39]. Mutations or deletions of a NxLxΦE motif [30] or an LPE motif [39] in human Scc1 result in drastically reduced cleavage efficiency [39]. Notably, these motifs are also present in the pseudosubstrate securin (see below). The spacer sequences between these motifs vary in sequence and length between securin and Scc1 and seem to play an important role in promoting efficient cleavage of human Scc1 [30,39]. Furthermore, these motifs help determine the antiparallel binding mode relative to separase (Figures 2a and 3b).

Studies in yeast [40] and mammals [10–12,15,41] suggest that phosphorylation is a conserved mechanism to promote the cleavage of some separase substrates. Calculation of the electrostatic surface potential of human separase reveals the presence of three positively charged patches (patches I–III) (Figure 3a). Interestingly, some substrate phosphorylation sites that enhance cleavage are located just a few residues N-terminal of the cleavage site. In yeast Scc1 [40] and human Meikin [11], phosphorylation of a serine residue at the P6 position is thought to promote cleavage. In other substrates, or the pseudosubstrate securin, this P6 Ser residue is replaced by an aspartate or a glutamate residue, thereby mimicking phosphorylation [33]. In all these cases, enhanced binding to separase is likely recognized through patch III adjacent to the active site. The crystal structure of the *Chaetomium thermophilum* separase protease domain bound to a phosphorylated Scc1 substrate peptide provides clear structural evidence for the interaction of phosphorylated substrates at the P6 position with patch III [31].

In some separase substrates, such as Pericentrin [15] and Rec8 [42], there is evidence that cleavage is enhanced by phosphorylation at multiple sites that are more distant from the cleavage site. We speculate that more distant phosphates interact with patches I and/or II, or possibly at smaller basic sites on the separase surface.

Using human securin and Scc1 AlphaFold2 [43] predictions as templates to predict the potential binding path of substrates, it seems plausible that patch II (Figure 3a) serves as a recognition site for phosphorylated residues that, in some substrates, are located 10–40 aa C-terminal of the P1 site. This positively charged region located in the TPR-like domain of separase has recently been described as a substrate-binding site in yeast separase (also known as Esp1) [44]. In the human separase–securin complex, glutamates 132 and 133 of securin form salt bridges with arginine 947 and lysine 944, part of patch II, respectively. Glutamate 132 is part of the aforementioned LPE motif and is recognized by patch II (PDB code: 7NJ1 [30]). In yeast, phosphorylation of securin (also known as Pds1) at serine 277, serine 292 and threonine 304 facilitates efficient interaction with separase [45]. Modeling of phosphoserine 277 in the Esp1-Pds1 crystal structure (PDB code: 5U1T [32]) reveals the potential formation of salt bridges of pSer 277 with Arg798 and/or Arg 1130, both involved in forming patch II.

Another distinct positive patch (patch I) is situated in the N-terminal HEAT-repeat domain. However, structural or biochemical data that substantiate a role of patch I in the recognition of phosphorylated substrates do not exist to date. Furthermore, all three patches may be involved in the binding and stimulation of cleavage activity by DNA [39,46].

## Modes of separase inhibition by securin or the CDK1–cyclin B complex

The determination of structures of separase bound to securin [30,32,33] or the CCC complex [30] have provided unexpected molecular insights into two fundamentally different modes of separase inhibition. Securin binds to separase as an extended, antiparallel pseudosubstrate (Figure 2a) that distorts the catalytic site geometry by replacing the invariant P1 arginine with a large hydrophobic residue. The insertion of a hydrophobic residue into the catalytic pocket leads to an outwards rotation of the aforementioned invariant aspartate residue in separase (Asp2151 in *C. thermophilum* [31], Asp1080 in *Caenorhabditis elegans* [33] and Asp2070 in *Homo sapiens* [30]), as well as the disruption of the hydrogen bonding network, which ultimately causes the displacement of the catalytic histidine residue by almost 2 Å and thus prevents cleavage of the scissile peptide bond in the inhibitory pseudosubstrate securin [33]. Consistent with these findings, the artificial introduction of an arginine residue at the P1 position leads to the transformation of securin into a cleavable substrate [31,39,47]. High-affinity binding between securin and separase is furthermore accomplished through additional substrate-binding sites in separase (exosites) that span the entire enzyme [39]. Securin contains multiple regions that are crucial for efficient inhibition, including the NxLxΦE and LPE motifs as well as a stretch of hydrophobic residues that follows these motifs (aa 136–146) [30,39].

In contrast, the CCC complex binds to separase at a distinct binding site in an interdomain cleft between the central TPR-like domain and the C-terminal protease domain. Stable association between the CCC complex and separase strictly depends on the phosphorylation of serine 1126 of human separase by CDK1 [25,30]. CCC binding causes the rigidification of several separase loop segments, including insert 1 and parts of insert 2. Upon phosphorylation, the intrinsically disordered insert 1 wraps around cyclin B1, with phosphoserine 1126 at its center (Figures 1a,b and 2b). The phosphoserine is recognized by a highly conserved, positively charged phosphate-binding pocket present in B-type cyclins but not other cyclins. Interestingly, mutation of the homologous pocket of yeast Clb2 leads to reduced phosphorylation of the CDK1 substrate Ndd1 [48], implying a broader regulatory role for this binding pocket in the recognition of CDK1 targets.

Other loop fragments that are rigidified upon CCC binding interact with substrate-binding sites to occlude substrate access and thus inhibit separase activity. We, therefore, termed these three loops auto-inhibitory loops or AILs [30]. AIL1 contains the aforementioned NxLxΦE motif, which is part of a short α-helix that fills an interdomain cleft between the central TPR-like domain and the C-terminal protease domain, adjacent to the active site (Figures 2b and 3b). Unpublished data from our laboratory suggest that this loop also binds this cleft in the apo form of separase, although the loop, in this case, exhibits a higher degree of flexibility, reflected by a less-well-defined density compared with that seen in the separase–CCC complex (data not shown). It is, therefore, possible that this loop modulates substrate recognition by active separase.

AIL2 is spatially closer positioned to the N-terminus of separase than AIL1 (Figure 1b) and blocks access to a hydrophobic channel near patch II in the TPR-like domain; this channel is occupied by a hydrophobic segment of securin in the securin–separase complex.

AIL3 is part of the large insert 2 and binds adjacent to the active site, opposite AIL1 (Figures 2b and 3b). Asn1394, Phe1395 and Ser1396 of AIL3 exhibit similarities to the DIE-motif in securin [47] (P6–P4 positions), while its carboxy-terminal acidic amino acids are recognized by the positively charged patch III [30]. AIL1 and AIL3 thus bind adjacent to both sides of the catalytic pocket to occlude substrate binding (Figure 2b). Removal of AIL1 or AIL3 increases catalytic activity, as expected for an inhibitory role of these loop segments [30]. Insert 2 also contains a CDC6-like motif that binds and thereby inhibits CDK1 [27,30] (Figure 2b). The result is a complex in which separase and CDK1 are caught ‘in an oppressive embrace’ [49].

It has been suggested that the third described inhibitor of separase, the SGO2–MAD2 complex, uses a non-cleavable pseudosubstrate sequence to occupy and inhibit the active site of separase, comparable to securin [28]. The proposed sequence motif is situated within a region of SGO2 that is predicted to form a coiled-coil domain [50], and therefore additional structural studies will be required to clearly define the mode of separase inhibition by the SGO2–MAD2 complex.

## Regulation of separase activity by PP2A

Stable association of securin or the CCC complex with separase is modulated by phosphorylation of the protease and its substrates, and the phosphatase PP2A is likely to play a role in these mechanisms. PP2A binding to separase is mediated through its B56 subunit [35,37,51,52]. The binding site of PP2A has been mapped inside insert 2 of separase, near the self-cleavage sites (Figure 1) [35]. Sequence analysis of PP2A-B56 interactors led to the assignment of an (L/F/M)xx(I/V/L)xE consensus sequence for PP2A-B56 binding proteins [53], with the glutamate being the only invariant residue. Through sequence analysis, we identified a matching MxxIxE motif within aa 1485–1490 of human separase that overlaps with the first self-cleavage site (Arg 1486, Arg1506, Arg1535 are the P1 residues for each of the three self-cleavage sites). In accordance with these findings, separase self-cleavage disrupts PP2A binding [35].

Self-cleavage of separase promotes the binding of cyclin B1 to separase *in vivo* [37] but is not required for the assembly of the purified CCC complex *in vitro* [30]. One potential explanation is that associated PP2A *in vivo* dephosphorylates Ser1126, the site required for cyclin B association. According to this model, self-cleavage enhances separase phosphorylation and cyclin B association by disrupting PP2A binding.

The effect of securin phosphorylation on its binding to separase is species- and position-dependent. Phosphorylation of C-terminal residues (relative to the pseudo-cleavage site) in yeast securin Pds1 promotes interaction with yeast separase Esp1 [45], perhaps through interaction with the positively conserved charged patch II. Dephosphorylation of N-terminal phosphosites (relative to the pseudo-cleavage site) in human securin by associated PP2A stabilizes separase-bound securin because it delays APC/C-dependent ubiquitylation [51]. The association of securin with PP2A is indirect and bridged by the separase–PP2A interaction [51].

## Conclusions

The proteolytic activity of human separase is regulated through an intricate and multi-layered interplay of diverse binding partners that modulate enzyme activity by influencing binding to substrates [10–12,15] or the pseudosubstrate securin [45,51].

Handover models for separase regulation and their implications in sister chromatid separation have been proposed [52,54]. In such models, separase is initially inhibited by securin that binds to separase co-translationally and acts as a chaperone to promote separase folding. Separase is then ‘handed over’ to other regulators. During metaphase, securin and cyclin B are degraded with similar kinetics via APC/C-mediated ubiquitylation. Securin destruction leads to activation and self-cleavage of separase, which in turn promotes the dissociation of PP2A. Reduced levels of bound PP2A may have a dual effect. First, any remaining phosphorylated securin that rebinds active separase will not be protected from APC/C-mediated destruction, thereby increasing the population of active separase [51]. Second, reduced PP2A binding to separase might also result in increased phosphorylation of separase Ser1126, which then promotes binding of a portion of the separase population to CDK1–cyclin B complexes that have not yet been inactivated via the APC/C [37]. Both mechanisms, namely enhanced securin degradation and increased Ser1126 phosphorylation, favor the association of separase with CDK1–cyclin B over securin and may be sufficient to establish a handover mechanism. It also remains possible that some securin destruction occurs at low rates earlier in mitosis, allowing the formation of separase–cyclin B complexes prior to metaphase.

Formation of the separase–CCC complex leads to mutual repression of the protease activity of separase and the kinase activity of CDK1. It has long been known that efficient poleward movement of separated sister chromatids depends on the shutdown of CDK1 activity [55–57]. Separase links these two independent processes of cohesin dissolution and segregation of the sister chromatids through the diverse action of its two inhibitors [54]. It remains unclear how the SGO2–MAD2 complex is integrated into this regulatory system, or whether it also binds to separase that is initially occupied by securin [28].

While recent structural and functional studies have begun to shed light on the diverse molecular mechanisms that control substrate recognition and human separase inhibition, much remains to be learned about this fascinating key player of cell division. For example, the structure of a full-length substrate bound to separase will likely provide novel insights into substrate recognition. Similarly, a complex structure of separase bound to PP2A will potentially offer novel insights into separase regulation by phosphorylation.

## Perspectives

- Equal distribution of the duplicated genetic material during mitosis is key for the genesis of healthy eukaryotic cells. Separase-mediated sister chromatid separation is a key step in this process.
- The proteolytic activity of separase is tightly controlled through binding partners that inhibit enzyme activity by blocking substrate binding. Vertebrate separase is kept in check by the action of two complexes whose inhibitory mechanisms fundamentally differ from each other.
- Structural and functional studies of separase bound to substrates and/or other regulatory binding partners will provide further insights into the molecular mechanisms that underlie chromosome segregation.

## Open Access

Open access for this article was enabled by the participation of the University of Geneva in an all-inclusive *Read & Publish* agreement with Portland Press and the Biochemical Society under a transformative agreement with Individual.

## Abbreviations

CCC: CDK1–cyclin B1–Cks1
CDK1: cyclin-dependent kinase 1
HEAT: huntingtin, elongation factor 3, protein phosphatase 2A, and TOR1
PP2A: protein phosphatase 2A
TPR: tetratricopeptide repeat
